# Introducing the brain erythropoietin circle to explain adaptive brain hardware upgrade and improved performance

**DOI:** 10.1038/s41380-022-01551-5

**Published:** 2022-04-12

**Authors:** Hannelore Ehrenreich, Laura Fernandez Garcia-Agudo, Agnes A. Steixner-Kumar, Justus B. H. Wilke, Umer Javed Butt

**Affiliations:** Clinical Neuroscience, Max Planck Institute for Multidisciplinary Sciences, City Campus, Göttingen, Germany

**Keywords:** Psychiatric disorders, Diseases

## Abstract

Executive functions, learning, attention, and processing speed are imperative facets of cognitive performance, affected in neuropsychiatric disorders. In clinical studies on different patient groups, recombinant human (rh) erythropoietin (EPO) lastingly improved higher cognition and reduced brain matter loss. Correspondingly, rhEPO treatment of young rodents or EPO receptor (EPOR) overexpression in pyramidal neurons caused remarkable and enduring cognitive improvement, together with enhanced hippocampal long-term potentiation. The ‘brain hardware upgrade’, underlying these observations, includes an EPO induced ~20% increase in pyramidal neurons and oligodendrocytes in cornu ammonis hippocampi in the absence of elevated DNA synthesis. In parallel, EPO reduces microglia numbers and dampens their activity and metabolism as prerequisites for undisturbed EPO-driven differentiation of pre-existing local neuronal precursors. These processes depend on neuronal and microglial EPOR. This novel mechanism of powerful postnatal neurogenesis, outside the classical neurogenic niches, and on-demand delivery of new cells, paralleled by dendritic spine increase, let us hypothesize a physiological procognitive role of hypoxia-induced endogenous EPO in brain, which we imitate by rhEPO treatment. Here we delineate the *brain EPO circle* as working model explaining adaptive ‘brain hardware upgrade’ and improved performance. In this fundamental regulatory circle, neuronal networks, challenged by motor-cognitive tasks, drift into transient ‘functional hypoxia’, thereby triggering neuronal EPO/EPOR expression.

## The particular situation of contemporary research on erythropoietin

Recombinant human (rh) EPO has been an approved and safe drug for treating renal anemia for meanwhile over 35 years [1–5]. Over the last 25 years, roles of EPO and its receptor, EPOR, in brain became increasingly evident [6, 7], even though the existence of an endogenous brain EPO system has for long time remained a matter of dispute [8]. Explaining the multifaceted brain EPO system, however, will be pivotal to understand adaptive brain mechanisms and develop novel treatment strategies for brain diseases, exploiting both, rhEPO and hypoxia-induced brain EPO. This in turn faces several challenges.

The rhEPO market has always been highly lucrative, but patents expired around 2008. Industry, facing competition by biosimilar producers and fearing off-label use and emergence of new side effects, withdrew essentially all support of research on further rhEPO indications, while funding agencies keep sending applicants for clinical EPO projects to industry. Its name, derived from the original description in erythropoiesis and somewhat misleading for neuroscientists or reviewers, and its negative reputation as doping drug have not helped funding translational research on EPO. This is unfortunate considering that >300 preclinical studies since 1998 showed beneficial effects of high-dose rhEPO in a wide spectrum of brain disease models [6, 7, 9–12].

**Fig. 1:**
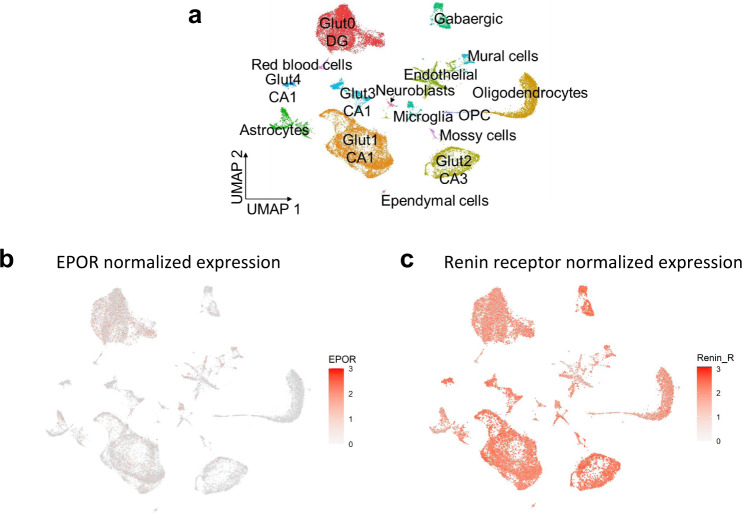
Dropout effect in single-cell transcriptome analysis. **a** Unbiased clustering of hippocampal cells from mice under normoxia (*n* = 2) or hypoxia (*n* = 2), represented in UMAP space, reveals 16 distinct cell populations across conditions [77]. **b** The extremely low expression of EPOR in these populations, compared to the abundant expression of renin receptor (**c**), illustrates the partial ‘dropout effect’ of scRNA-seq for EPOR. We note that EPO is not detectable at all using this method.

In fact, considerable upregulation of EPO/EPOR in brain occurs for instance upon brain injury [7, 13], which stimulated researchers early to explore the neuroprotective and neuroregenerative potential of this growth factor [9, 14]. Notwithstanding all this evidence, underlying mechanisms have remained widely obscure. Even though research on extra-hematopoietic, direct tissue-protective properties of rhEPO started in the nineties [9, 14–16], results were partly controversial [8, 13], owing to lack of necessary tools, e.g., specific antibodies or targeted genetic models. But also the extremely low brain expression of EPO/EPOR poses a huge problem (Fig. 1), with EPO undetectable by e.g., single cell RNA sequencing (scRNA-seq) - a so-called ‘drop-out effect’ across tissues - and EPOR even on ‘strongly expressing’ hematopoietic cells amounting to much less than 1000 molecules per cell [13, 17]. At the same time, this low expression reflects perfectly the incredible potency of this growth factor.

## Unusual reverse translation in brain EPO research: Human trials first

Following own clinical observations as neurologist and psychiatrist (HE), starting over 30 years ago, and contrary to the usual translational methodologies in neuroscience, we started with investigator-initiated clinical trials on rhEPO already in 1998, including trials in ischemic stroke, multiple sclerosis, schizophrenia, major depression and bipolar disease [18–23]. This ‘human trials first’ approach led to the discovery of the highly reproducible neuroprotective and procognitive effects of rhEPO in humans, independent of hematopoiesis. In addition, it documented repeatedly a significant reduction of gray matter loss in brain disease upon rhEPO treatment [24–26]. Strikingly, in healthy humans, application of a single high-dose of rhEPO already increased the hippocampal response during memory retrieval [27].

Many effects of rhEPO treatment on cognition, neuroprotection, and neuroregeneration, however, have for a long time been difficult to persuasively separate from rhEPO effects on erythropoiesis/hematocrit. Acute anemia, for instance, affects human brain functions and respective neurophysiological measures like P300, while anemia correction or oxygen breathing results in prompt improvement [28].

## Separating EPO effects on brain from hematopoiesis

An elegant study employing either transgenic mice with EPO overexpression exclusively in brain or wildtype mice receiving just one high-dose rhEPO injection showed convincingly that EPO in brain has remarkable effects on exercise performance in absence of any hematocrit changes [29]. This can for instance explain the intriguing brain effects of athletes upon EPO doping, e.g., increased drive and motivation [30]. Even though doping in sports is definitely not reinforced here, it represents one of the most convincing ‘field studies’, supporting the strong and enduring effects of rhEPO on brain performance.

Substantial additional evidence supports that EPO actions in brain are not related to hematopoiesis: (i) There was either no increase in hematocrit or no correlation between such increase and cognition. (ii) The effect on cognition by far outlasted any transient effect on hematocrit [18, 20, 22, 26, 31]. (iii) Even more compellingly, the above-cited study on transgenic EPO expression in brain [29] and (iv) non-hematopoietic EPO analogues [32–37] have been further key arguments for EPO actions on brain in absence of blood effects. (v) Moreover, boosted cognition and neuroplasticity of mice expressing constitutively active EPOR in glutamatergic pyramidal neurons [38], but not in GABAergic interneurons [39], suggested a central role of these neurons for EPO effects on cognition, independent of hematopoiesis.

## Back-translation of brain EPO research to mice: procognitive effects

With sophisticated higher cognition testing in normal mice, employing the *Five Choice Serial Reaction Time Task* [40, 41] or visual discrimination tasks in the *Touch-Screen* based operant system [42], we initiated targeted ‘back-translational’ work. We were able to document faster, more pronounced and lastingly improved attention, learning and memory, together with enhanced hippocampal long-term potentiation upon rhEPO application [43–45]. Similar cognitive improvement ensued from experiments using prolylhydroxylase inhibitors for stabilization of hypoxia-inducible factor (HIF). This HIF stabilization, leading to increased EPO expression in brain (and other tissues), provided first hints that endogenous EPO may be sufficient to improve cognition, but did not ultimately prove it, since vascular endothelial growth factor, for instance, also rises upon HIF stabilization [46, 47] and can enhance hippocampal learning and memory [48].

## Brain EPO adds a new layer of complexity to postnatal neurogenesis

**Fig. 2:**
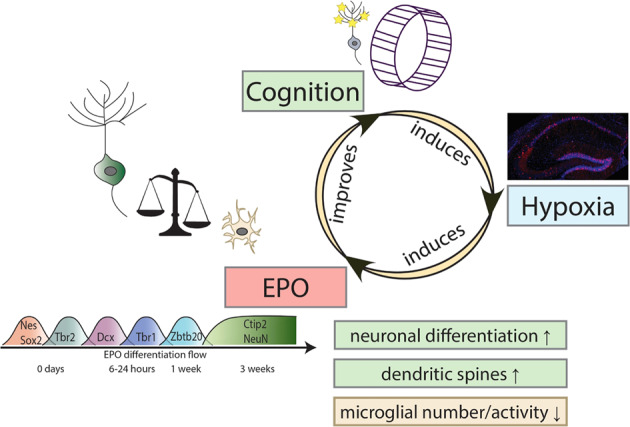
The brain EPO circle. Focusing on cornu ammonis (CA) pyramidal neurons, we delineate here a physiological circle of enduring neuroplasticity through enhanced dendritic spine density and swift generation of new functional neurons from diverse precursors without proliferation. Apparently, the entire precursor cell lineage in adult murine CA that is ready to differentiate towards pyramidal neurons remains ‘in flow’. In the proposed neuronal lineage progression, the EPO-responsive progenitor cells and immature neurons may never constitute abundant clusters in a cross-sectional steady-state analysis, but increases rather occur in transient waves with individual neurodifferentiation markers just rising at particular time windows. In this process, neuron-microglia counterbalance plays a pivotal role with both microglial and pyramidal neuronal EPOR being critical for neuronal differentiation upon EPO. Elimination of the pyramidal neuronal EPOR eradicates EPO-driven neurodifferentiation. Strikingly, also upon microglial EPOR deletion, the acceleration by EPO of neuronal differentiation is abolished. We note that the brain EPO circle can be entered anywhere, starting either with mild to moderate inspiratory hypoxia, with rhEPO treatment or with motor-cognitive challenge as inducer of functional hypoxia. Under all these circumstances, brain EPO (and EPOR) emerge as central players of a novel mechanism driving neuronal differentiation and lasting plasticity [54, 77, 105]. *Note: In this sketch, the balance symbolizes cell numbers and activity, not weight, i.e., microglia numbers/activity go down while pyramidal neuron numbers go up* [105].

Some of the neuroprotective and neuroregenerative actions of rhEPO in disease models were speculated to be related to neurogenesis [49–52], but for a long time, no data were available to support this hypothesis or to discriminate between rhEPO effects on neural progenitor proliferation versus differentiation in postnatal brain [51]. In hematopoiesis, EPO is crucial for antiapoptotic effects on erythroid precursors and for their differentiation rather than for proliferation [53]. We therefore asked whether similar mechanisms apply for the brain. Based on our findings of EPO enhancing cognition and long-term potentiation [43], we initiated our recently published studies on healthy young mice, in which we investigated the effects of three-week administration of rhEPO, starting at the age of four weeks, on brain cell numbers [45, 54]. Entirely unexpected was the discovery that this treatment leads to an around 20% increase in numbers of pyramidal neurons in cornu ammonis (CA), in absence of any respectively altered cell proliferation or apoptosis [45]. Using nanoscopic secondary ion mass spectrometry (NanoSIMS), we found that in rhEPO-treated mice, fed with 15N-leucine diet, an equivalent ~20% of neurons revealed elevated 15N-leucine incorporation, reflecting high de-novo protein synthesis [45]. Under constant cognitive challenge (but not without), the discovered increase in neuron numbers persisted until over six months of age [45]. Reassuringly, the increase in pyramidal neuron numbers and dendritic spines is similarly observed when EPO treatment starts at older age, precluding a purely late developmental phenomenon [54]. We concluded that at any age, EPO most likely drives the differentiation of diverse non-dividing, pre-existing local precursors within neuronal lineages, i.e., in absence of elevated DNA synthesis. Uncovered by serendipity with rhEPO, our observations added a new layer of complexity to postnatal neurogenesis and neuroregeneration where brain expressed EPO appears to play a crucial role in the sense of a ‘neurogenesis fast track’. This novel role of brain EPO is best sketched with a circle including motor-cognitive challenge and ‘functional hypoxia’ (see below; Fig. 2).

## Functional hypoxia as part of a novel model of neuroplasticity

Hypoxia is the term for reduced oxygen in cells/tissues, relative to ‘normal’. Earlier interpreted as principally pathological, e.g., upon cardiac arrest, hypoxia is increasingly recognized as strong driver of development including angiogenesis, hematopoiesis, or regeneration [55–59]. In perfect support, quite some literature deals with hypoxic/ischemic preconditioning, where sub-lethal insults induce protection against later fatal injuries by improving tolerance, e.g., through lasting or stand-by activation of hypoxia signaling pathways, anti-apoptosis, anti-excitotoxicity, and autophagy [60]. Noteworthy, hypoxia can be the ultimate consequence of various processes, including lower oxygen availability due to hypoxemia, anemia, perfusion impairment, or increased oxygen consumption (‘functional hypoxia’). Although the mere cellular responses to physiological and pathological hypoxia are similar, they are accompanied by different parallel mechanisms, potentially directing responses towards beneficial effects as observed upon mild to moderate inspiratory hypoxia or functional hypoxia (as discussed in this review) or adverse effects, e.g., occurring upon ischemia or dysregulation of cerebral perfusion [61].

In 2019, W.G. Kaelin, P.J. Ratcliffe & G.L. Semenza jointly received the Nobel Prize in Physiology and Medicine for their discoveries of how cells sense and adapt to oxygen availability [55–59]. The hypoxia-induced transcriptional program is partly independent of [62–64] and partly controlled by HIF, binding to hypoxia-responsive elements to modulate gene expression, including EPO [6, 29, 46, 60, 63, 65–69]. This fundamental context, however, has not been systematically translated to normal brain functions, where it likely has a central, yet unheralded role. Extensive physical activity and cognitive challenge lead to widespread brain activation and improved global brain function including mood [70, 71]. Neurologists/psychiatrists encourage patients to improve functions by practicing: *‘Use-it-or-lose-it’*. Hippocampal volume increases upon exercise in healthy or schizophrenic subjects, correlating with e.g., improved short-term memory [72]. We hypothesized that activity-induced, ‘functional hypoxia’ of neurons and brain-expressed EPO may play pivotal roles in all these circumstances. But this had still to be proven as detailed below. In perfect agreement with this concept, but without even considering functional hypoxia as the driving force, a recent elegant study on a mouse model of Rett syndrome showed that intensive physical training, beginning in the presymptomatic period, dramatically improves the performance of specific motor and memory tasks, and significantly delays the onset of symptoms. Task-specific neurons that are repeatedly activated during training were found to develop more dendritic arbors and to have better neurophysiological responses than those in untrained mice [73]. Rett syndrome is a progressive neurological disorder in which children develop normally for the first one or two years of life, before experiencing profound motor and cognitive decline. The results of this beautiful work strongly encourage novel therapeutic approaches in Rett syndrome but also in other neurodevelopmental conditions like many forms of intellectual disability or (syndromal) autism, as for instance in fragile X syndrome or *SYNGAP* mutations [74–76]. These novel therapeutic approaches likely build on functional hypoxia as pivotal driving force.

## The brain EPO circle explaining adaptive brain hardware upgrade and enhanced performance

**Fig. 3:**
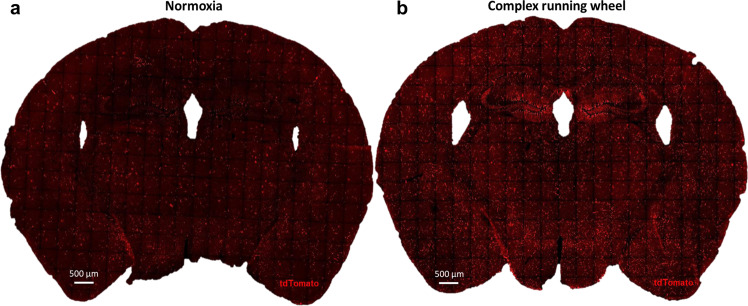
Labeling hypoxic cells. Illustrative coronal brain sections of *CAG-CreERT2-ODD::R26R-tdTomato* mice under (**a**) normoxia and (**b**) voluntary complex running wheel performance *(‘functional hypoxia’)*. Motor-cognitive challenge (over 5 consecutive days with daily tamoxifen injections, 5 in total) induces globally enhanced numbers/intensity of tdTomato+ cells in the brain with particular focus on hippocampus and cortex (pyramidal neurons) [77].

With a refined transgenic reporter approach, utilizing the oxygen-dependent degradation (ODD) domain of Hif-1α fused to CreERT2 recombinase, *CAG-CreERT2-ODD::R26R-tdTomato* mice [77, 78], we provided first clues that complex running wheel (CRW) exposure as voluntary motor-cognitive challenge leads to ‘relative oxygen deficiency’ in hippocampal pyramidal neurons, i.e., initially higher oxygen consumption than delivery, which we coined ‘functional hypoxia’. Figure 3 shows tdTomato+ hypoxic cells in representative mouse brain sections. We also spatially mapped these cells by light-sheet microscopy [77]. This three-dimensional view allowed to demonstrate hypoxia across essentially all brain areas. Amazingly, running on CRW causes hypoxic neurons all over, and particularly abundant in hippocampus. Here, the CRW-induced endogenous hypoxia of pyramidal neurons augments EPO and EPOR expression, as experimentally proven by in situ hybridization [54]. EPO and EPOR in turn prompt via auto/paracrine signaling the emergence of new pyramidal neurons and, in parallel, enhance dendritic spine densities of preexisting neurons. As a result, performance is improved, conveniently referring to ‘EPO brain doping’ [54].

An explanation for the fast appearance of a substantial amount of new mature pyramidal neurons upon EPO in just 3–4 weeks was provided by scRNA-seq in CA1, revealing an increase in newly differentiating neurons already 6 h after a single intraperitoneal rhEPO injection. This means that we see the start of enhanced differentiation already at this early time point, as illustrated by a pseudotime presentation of the trajectory analysis of neuronal cells, performed in *Monocle2* [54, 79]. In fact, the increase in the number of differentiating immature neurons starts at 6 h after the first EPO injection with an increase in T-box brain1 (Tbr1) positive neuronal precursors over the placebo condition (Fig. 2). Additional markers that characterize this early responding cluster include doublecortin (Dcx) and transducin-like enhancer family member4 (Tle4). At one week after EPO treatment start, we see an increase in a subsequent differentiation stage (Zbtb20 positive immature neurons). This EPO-induced differentiation flow is ultimately leading after 3–4 weeks to a 20% increase in mature pyramidal neurons with expression of the markers Ctip2/NeuN, while the other transcripts/proteins are no longer increased under EPO but have returned to control/placebo level. This rapid, wave-like drive of neurodifferentiation was similarly observed upon hypoxia-induced expression of EPO in brain. Exposure to moderate inspiratory hypoxia (12% O_2_) imitated the improved CRW performance as well as the enhanced neuron numbers and dendritic spines upon rhEPO, and inspiratory hypoxia in combination with CRW (*add-on* endogenous, functional hypoxia) even acted synergistically. All these effects depend on pyramidal neuronal expression of the EPOR gene [54].

Taken together, an intriguing novel model of neuroplasticity emerged, in which specific task-associated neuronal networks drift into transient functional hypoxia as a local as well as a brain-wide response comprising indirectly activated neurons and non-neuronal cells [77, 80]. This in turn triggers neuronal EPO/EPOR expression to mediate neuroplasticity via adaptive transcript regulation in the behaving brain, leading to substantial ‘hardware upgrade’ (Fig. 2).

## Effects of pharmacological oxygen manipulations on brain function

We are just starting to understand the role of oxygen manipulations in the brain with respect to effects on brain performance and brain disease. Even though not directly related to the main topic of this perspective, we note that a whole array of different methods on modulating oxygen availability have been described to influence brain functions in health and disease models. Just examples are recent work on hyperbaric oxygen therapy that reported alleviation of vascular dysfunction and amyloid burden in an Alzheimer’s disease mouse model and also in elderly patients [81]. Similarly, intermittent hypoxic–hyperoxic training in geriatric subjects [82] and in patients with mild cognitive impairment [83] showed beneficial effects on cognition. In this context, the endogenous EPO system likely plays an important role. In fact, a coding single nucleotide polymorphism of EPO, the nonsynonymous rare variant SNP rs62483572, was identified to be protective in Alzheimer’s disease [84]. Moreover, human EPO and EPOR *gain-of-function* genotypes are associated with better cognition [85].

Also upon chronic moderate hyperoxia, increased HIF accumulation and EPO expression in mouse brain have been reported [86], pointing to some overlap in downstream mechanisms of hypoxia and hyperoxia, reflected or mediated e.g., by excess reactive oxygen species (ROS, see below) [87–89]. On the other hand, contrasting effects of hypoxia versus hyperoxia were found in a mouse model of Friedreich’s ataxia where breathing of 11% O_2_ attenuated the progression of ataxia, whereas breathing 55% O_2_ hastened it [90]. We conclude here, that much work needs to be done to understand respective mechanisms and to further exploit them for the benefit of patients. Overall, however, we should keep in mind, that hyperoxia is never physiological, whereas (functional) hypoxia definitely represents a major driving force of neurodevelopmental processes and adaptive neuroplasticity. Using hypoxia or rhEPO as treatment strategies, we imitate physiology, whereas hyperoxia would constitute some other sort of a pharmacological therapy. Nevertheless, any approach that demonstrates clear benefit in thus far untreatable diseases or syndromes should be considered and encouraged to be pursued. Even in COVID-19, where oxygen treatment depicts a logical and important therapeutic contribution, rhEPO treatment as substitution therapy might help overcome the unexpected ‘hypoxia paradox’ in this condition [91, 92].

Also worth mentioning in this context is the tight regulation between oxidative stress and antioxidants relating to EPO pathways. Strong intermittent hypoxic conditions (nadir of 5% O_2_), for instance, induce increased ROS production, which activate HIF. Of note, this strong hypoxia may damage parvalbumin-expressing interneurons through ROS accumulation, which apparently can be ameliorated by environmental enrichment [93]. On the other hand, ROS and antioxidants were shown to play a role in neuronal and oligodendroglial differentiation [94–96]. Therefore, yet to be elucidated downstream mechanisms of hypoxia and EPO may involve some antioxidant compensation to balance ROS increase and to allow undisturbed cellular differentiation processes.

## Delineating the multifaceted and multicellular brain EPO system

Importantly, effects of EPO on cognition and neuroregeneration comprise parallel effects on other cell types. In a mouse model of traumatic brain injury, we saw that early treatment with rhEPO prevented consequences of secondary neurodegeneration, i.e., progressive brain atrophy and cognitive decline [25, 97], reduced the increase in injury-induced microglia and dampened their motility [98, 99]. Reflecting the high - but still poorly understood - complexity of the brain EPO system, also interneurons [39, 100], oligodendrocytes, their precursors (OPC) [45], astrocytes, endothelial cells and pericytes express EPO and EPOR, at least in disease [6, 10], and EPO not only traverses the blood-brain-barrier (BBB), but influences BBB function, immune cell transmigration, angiogenesis, and cerebral blood flow [31, 101–103].

In fact, EPO effects on interneurons are still much less clear. EPO treatment decreases the structural complexity of certain interneuronal subpopulations and the density of inhibitory perisomatic puncta that surround pyramidal neurons *(Curto et al, manuscript in preparation)*. In a transgenic mouse line that constitutively overexpresses neuronal EPO from early development on, stimulation of postnatal GABAergic maturation and an elevation of hippocampal GABA-immunoreactive neurons was reported, together with increase in interneurons expressing parvalbumin, somatostatin and neuropeptide Y [100]. Whereas constitutively active EPOR in GABAergic neurons changed hippocampal network properties, cognition was not affected, suggesting that the effect of EPO on cognition is dominated by its effect on the glutamatergic system [39]. Much more work is needed to fully understand the impact of EPO on the inhibitory circuitry.

In EPO-treated NG2-CreERT2 mice, we confirmed enhanced differentiation of pre-existing oligodendrocyte precursors (NG2+), again in absence of elevated DNA synthesis [45]. Completely unexpected was the observation that mice with lack of oligodendroglial EPOR apparently develop a mild dementia phenotype, as demonstrated in our IntelliCage test paradigm [104]. This paradigm allows cognitive, emotional and social phenotyping of mice in an observer-independent setting. The underlying mechanisms of this OPC/oligodendrocyte-related cognitive/intellectual disability are not yet understood and presently subject to extensive study in our laboratory.

An unforeseen involvement of resident microglia started with the discovery that stimulated neurodifferentiation, either by rhEPO, functional or inspiratory hypoxia, goes hand in hand with a decrease in microglia numbers. Obviously, during accelerating neuronal differentiation, EPO acts as regulator of the CSF1R-dependent microglia. Here, neuronally expressed IL-34 and microglial CSF1R are instrumental [105]. Surprisingly, EPO affects microglia in phases, initially by inducing apoptosis, later by reducing proliferation, and overall dampens microglia activity and metabolism, as verified by selective genetic targeting of either microglial or pyramidal neuronal EPOR. It turned out that EPOR on both cell types are critical for neuronal differentiation in CA upon EPO [105]. We note that the EPO effects on microglia are opposite to the effects on most other cell types, where this potent growth factor rather acts in a strong antiapoptotic fashion, enhances energy metabolism, and drives proliferation and/or differentiation. The deeper delineation of these contrasting cellular responses to the same ligand requires further study and may involve different EPORs or different EPOR properties as well as diverse downstream intracellular signaling.

## Conclusions and outlook

In summary, powerful, hematopoiesis-independent effects of rhEPO on neuroprotection, neuroregeneration and cognition in humans and rodents suggest that endogenous EPO in brain serves fundamental, previously overlooked physiological functions. The here introduced brain EPO circle explains as convenient working model the adaptive ‘brain hardware upgrade’ and enhanced performance upon rhEPO or brain EPO induction by hypoxia. In this fundamental regulatory circle, neuronal networks when challenged by motor-cognitive tasks, drift into transient ‘functional hypoxia’, thereby triggering neuronal EPO and EPOR expression (Fig. 2).

In other words, strong motor-cognitive exercise leads to neuronal activation and functional hypoxia, inducing HIF activation, followed by EPO transcription (among other transcripts) in pyramidal neurons, which in turn grow more dendritic spines and simultaneously stimulate their neighboring cells, ready to become neurons, to differentiate within the hippocampus. All this then contributes to cognitive improvement. We note, however, that the brain EPO circle can be entered anywhere, starting either with mild to moderate inspiratory hypoxia, with rhEPO treatment or with the aforementioned motor-cognitive challenge as inducer of functional hypoxia, leaving plenty of possible ways for future therapeutic interventions.

Remarkably, the bigger picture of the brain EPO system is still missing to date, despite supporting hints of hypoxia influencing cognition and of hypoxia-induced EPO [6, 7, 10]. All these hints are first fragments of a still concealed mosaic. Physiological conditions of brain EPO/EPOR expression, function and interplay of the different brain cell types with respect to the brain EPO system have remained widely unexplored. Published work on putatively different types of EPOR in brain is fragmentary and contradictory [106, 107]. Targeting EPO and hypoxia, the critical components of the here delineated regulatory brain circle, may yield promising innovative treatment approaches to neuropsychiatric diseases. But admittedly, for complete mechanistic understanding, much work still remains to be done.
